# Dilatometric Analysis and Kinetics Research of Martensitic Transformation under a Temperature Gradient and Stress

**DOI:** 10.3390/ma14237271

**Published:** 2021-11-28

**Authors:** Liheng Liu, Bin Guo

**Affiliations:** National Key Laboratory for Precision Hot Processing of Metals, School of Materials Science and Engineering, Harbin Institute of Technology, Harbin 150001, China; liuliheng0319@163.com

**Keywords:** dilatometric analysis, martensitic transformation kinetics, stress, temperature gradient

## Abstract

Based on material constitutive models and the classic Koistinen–Marburger (KM) kinetics model, a new dilatometric analysis model was developed to extract the kinetics curve of martensitic transformation under a temperature gradient and stress from the measured dilatometric data and to determine the transformation parameters. The proposed dilatometric analysis model is generally for athermal martensitic transformation, relying only on the average atom volume of martensite and austenite. Furthermore, through theoretical calculations, the proposed model also provided a more accurate method for obtaining the martensite start temperature, which is different from the traditional method. According to the dilatometric analysis results for the martensitic transformation of a type of high-strength low-alloy steel, and the thermodynamic basis of martensitic transformation, a refined kinetics model was developed that successfully predicted the martensitic transformation kinetics curves under different stresses, taking into account the physical significance of the transformation parameter α and the driving force of stress for martensitic transformation.

## 1. Introduction

The expansion of metal is essentially a continuous or discontinuous change in atomic volume caused by temperature change or phase transformation. This physical nature makes dilatometric analysis a powerful technique for studying the phase transformation behaviors in ferrous alloys [1,2,3].

The dilatometric data measured by a sensitive high-speed dilatometer can provide detailed information on the thermal expansion characteristics and the change in average atomic volume during transformation [4]. Using specific analysis models, the product phase fraction can be extracted as a function of temperature or time from the dilatometric curve.

**Figure 1:**
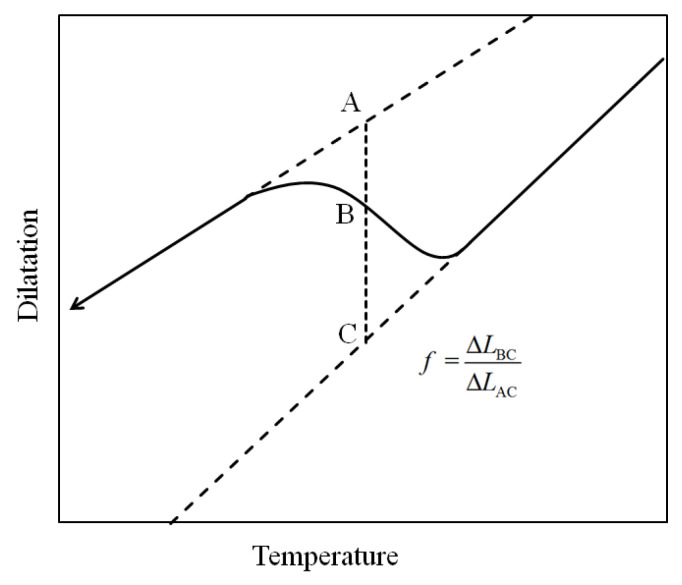
Schematic dilatometric curve during transformation.

The classic analysis model proposed to calculate the phase fraction from the dilatometric curve is the lever rule [5]. As shown in Figure 1, the linear expansion behaviors of the dilatometric curve are extrapolated into the temperature range, where phase transformation occurs. Assuming that the fraction of the product phase is proportional to the dilatation strain, at a given temperature, the fraction of the product phase can be calculated using Equation (1), according to the relative position of the dilatometric curve between the two baselines extrapolated from the linear segments:

(1) $$f=\frac{\Delta L_{BC}}{\Delta L_{AC}}$$

It should be realized that there are three implicit premises for the establishment of the lever rule model [1,4,6,7]:

(1) The transformation is essentially complete when the maximum strain of the dilatometric curve is reached, usually at room temperature.
(2) The lever rule can only be applied to single-phase transformation or to multiple phase transformations if they can be considered to be in sequence, with no overlaps.
(3) The lever rule is only valid for a transformation without repartition of alloy elements.

The previous premises limit the accuracy and availability of the lever rule in most materials, specifically in continuously-cooled steels after austenitizing. To overcome these shortcomings, dilatometric analysis models based on the average atom volume were developed to extract the transformation kinetics curve. Takahashi and Bhadeshia first examined the proportional relationship between the dimensional change and the fraction of product phase and provided a quantitative method related to lattice parameters [8]. Then Onink et al. conducted pioneering research in the quantification of simultaneous transformations [9,10]. The lattice parameters of austenite cementite and ferrite at elevated temperatures were measured by neutron diffraction and formulated as a function of carbon content. A numerical model was proposed to calculate the phase transformation kinetics curve of hyper-eutectoid Fe–C steel during an isothermal transformation by the formulated lattice parameters.

In subsequent studies, most of the researchers tried to expand the Onink model to a wider range of applications, while some researchers took a different approach, using the density of the constituting phases as the basis of their models [6,11,12]. Li et al. [13,14] suggested a dilatometric analysis model for the isothermal austenite decomposition in both hyper-eutectoid and hypo-eutectoid Fe–C steels. Some researchers took the effect of alloying elements on lattice parameters into account [15,16]. Garcia et al. [17] and Kop et al. [1] improved the model to analyze the transformations in continuously heating or cooling steels. In Kop’s study, the non-linear relationship between temperature and the atom volume of austenite due to the repartition of carbon was considered, which was normally neglected in the standard analysis of the dilatometric data.

The easily-ignored shortcoming of the average atom volume models, which did not consider the effect of the non-isotropic strain during transformation [4], was studied by Suh and Oh. In their study, the non-isotropic strain was attributed to the transformation plasticity, expressed as being proportional to the fraction of the product phase. In reference [18], they further distinguished the contribution of individual transformations to the evolution of non-isotropic dilatation and proposed a pair of linear relationships with different slopes.

The previous models aimed at the transformations without stress (mostly ferrite and pearlite transformations in steels, rather than martensitic transformation). However, martensitic transformation is essentially a stress-assisted transformation and stress can directly affect the kinetics, due to the stress-induced transformation. In addition, the mechanical behavior of the specimen is affected by stress during transformation, leading to transformation plasticity strain. Therefore, it is essential to develop an analytical model that takes the effect of stress into account.

Another easily overlooked fact is that the surface of the specimen [19], which is exposed to convective cooling, radiative cooling, and even stronger conduction cooling by mediums, can often be cooler than the core zone of the specimen. The temperature gradient can change the dilatometric curve significantly, through the pre-transformation on the cooler surface.

In the present paper, a new dilatometric analysis model was proposed to deal with the martensitic transformation under the function of temperature gradient and stress. By comparing the kinetics curves under different conditions, an improved kinetics model was developed that considers the physical significance of the parameter α and the effect of mechanical driving energy from stress.

## 2. Models

### 2.1. The Temperature Field and the Martensite-Start Temperature in the Specimen

**Figure 2:**
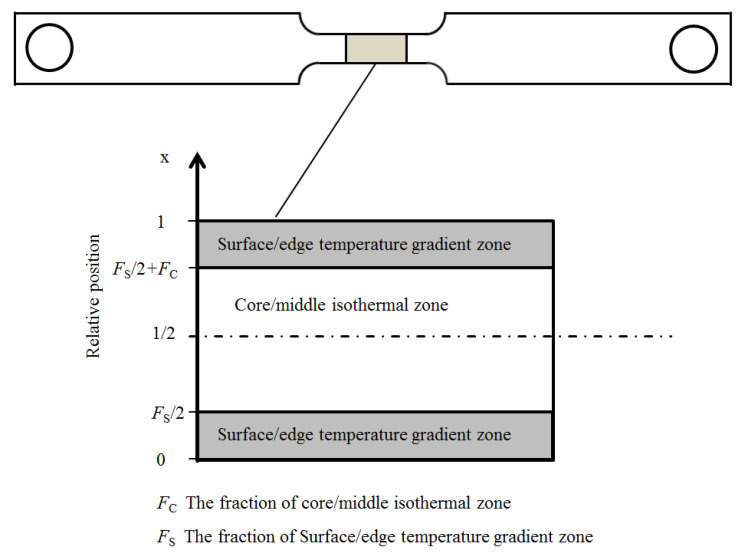
Schematic representation of temperature distribution in the specimen.

As shown in Figure 2, the transforming zone of the specimen for the Gleeble thermal-mechanical simulator in the present paper can be divided into two zones. Due to the position close to the thermocouples, the central/middle zone of the specimen can be regarded as an isothermal zone, since its temperature can be precisely controlled by the simulator. There is a temperature gradient in the surface/edge zone, due to stronger heat transfer.

Based on Fourier′s law and energy conservation law, the one-dimensional transient nonlinear differential equation along the transverse direction of the specimen can be expressed as [20]:

(2) $$\rho c\frac{\partial T}{\partial t}=\lambda \left(\frac{\partial^{2}T}{\partial x^{2}}\right)+q_{v}$$

where *t* is time, ρ is density, *c* is the specific heat capacity, λ is the heat transfer coefficient, and $q_{v}$ is the internal heat source, which can be expressed as the sum of the transformation latent heat $q_{1}$ and the heat from electric current $q_{e}$:

(3) $$q_{v}=q_{l}+q_{e}=q_{l}+\Delta Hdf$$

where ΔH is the enthalpy difference between martensite and austenite.

According to Equation (2), since the central/middle zone is an isothermal zone, the heat from the electric current can be expressed as:

(4) $$q_{e}=\rho c\frac{\partial T}{\partial t}-\Delta Hdf_{C}$$

When the temperature gradient in the surface/edge zone is small, Equation (2) can be approximated as:

(5) $$\rho c\frac{\partial T_{x}}{\partial t}=\lambda \left(\frac{\partial^{2}T_{x}}{\partial x^{2}}\right)+\rho c\frac{\partial T}{\partial t}+\Delta H\left(df_{S}-df_{C}\right)\approx \lambda \left(\frac{\partial^{2}T_{x}}{\partial x^{2}}\right)+\rho c\frac{\partial T}{\partial t}$$

where $T_{x}$ is the temperature at the point with the relative position x.

The boundary conditions in the dilatometric experiment can be expressed as:

(6) $$\left\{ \begin{array}{l} {T_{x}|}_{x=\frac{F_{S}}{2}}=T\\ {\lambda \left(\frac{\partial T_{x}}{\partial x}\right)|}_{x=0}=h\left(T_{0}-T_{s}\right)\approx h\left(M_{s}-T_{s}\right) \end{array}\right.$$

where $T_{s}$ is the ambient temperature, $M_{s}$ is the martensite-start temperature, *h* is the heat transfer coefficient of the surface, which can be approximated as a constant in a small temperature range.

Considering the symmetry, half of the specimen is taken as the research object. According to Equations (5) and (6), the integral calculation gives:

(7) $$T_{x}=\left\{ \begin{array}{l} T+\Delta T_{0}-\frac{2\Delta T_{0}}{F_{S}}x\;\left(0\leq x\leq \frac{F_{S}}{2}\right)\\ T\;\left(\frac{F_{S}}{2}\leq x\leq \frac{1}{2}\right) \end{array}\right.$$

where $\Delta T_{0}$ is the maximal difference of temperature between the central/middle zone and the surface/edge zone. With a small temperature gradient, due to less impact on kinetics curve, $\Delta T_{0}$ can be approximated as a constant during transformation and calculated by:

(8) $$\Delta T_{0}=-\frac{F_{S}}{2\lambda_{0}}Lh\left(M_{s}-T_{s}\right)$$

where *L* is the width of the specimen, $\lambda_{0}$ is the heat transfer coefficient of austenite at the reference temperature.

According to Equation (7), when $T_{x}=M_{s}$, the martensitic transformation starts at the point with the relative position x, and the martensite-start temperature $M_{\mathrm{sx}}$ measured by thermocouples in the core/middle zone can be expressed by:

(9) $$T=M_{\mathrm{sx}}=\left\{ \begin{array}{l} M_{s}-\Delta T_{0}+\frac{2\Delta T_{0}}{F_{S}}x\;\left(0\leq x\leq \frac{F_{S}}{2}\right)\\ M_{s}\;\left(\frac{F_{S}}{2}\leq x\leq \frac{1}{2}\right) \end{array}\right.$$

where $M_{s}$ is the martensitic transformation start temperature without stress.

Patel and Cohen [21] considered that the work done by stress contributed to the driving force of transformation and gave an expression for $M_{s}^{\prime }$, the transformation start temperature under tensile stress:

(10) $$M_{s}^{\prime }=M_{s}-\frac{dT}{d\Delta G^{\gamma \to \alpha }}U_{\max}^{\prime }\approx M_{s}-0.122\frac{dT}{d\Delta G^{\gamma \to \alpha }}\sigma_{1}$$

where $\Delta G^{\gamma \to \alpha }$ is the difference of Gibbs free energy between martensite and austenite, $U_{\max}^{\prime }$ is the maximum mechanical driving energy, and $\sigma_{1}$ is the tensile stress applied on the specimen.

**Figure 3:**
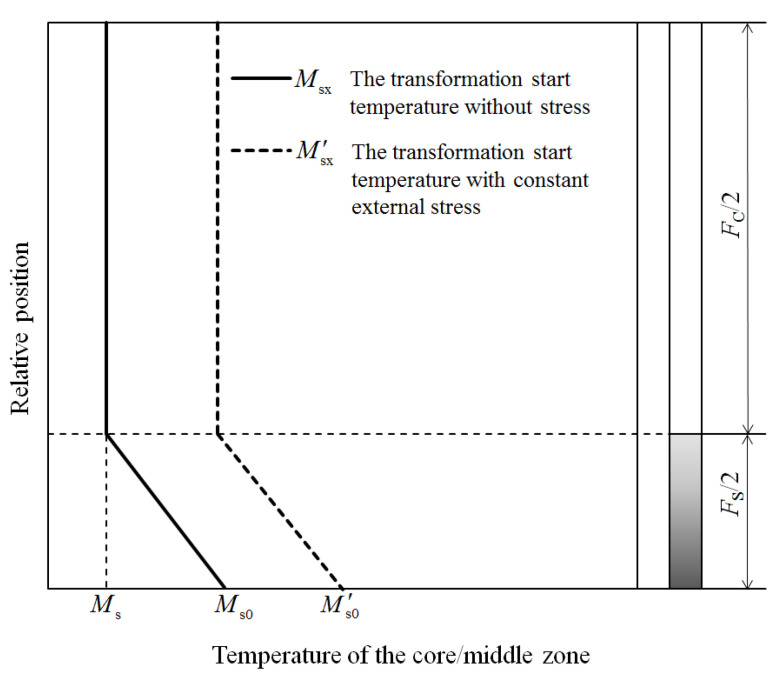
The transformation start temperature under a temperature gradient and stress.

Although the temperature gradient and the non-simultaneous martensitic transformation lead to internal stress in the specimen, the strain from transformation and transformation plasticity can rapidly reduce the internal stress and result in a uniform stress field in the complete specimen. Therefore, with a small temperature gradient, the martensite induced by internal stress can be ignored. Then, according to Equations (9) and (10), the martensite start temperature $M_{\mathrm{sx}}$ and the martensite start temperature under external stress are shown in Figure 3.

### 2.2. Extracting the Model of the Martensitic Kinetics Curve under a Temperature Gradient and Stress

During martensitic transformation under stress, the measured strain change Δε can be written as the sum of individual components, as the following [22]:

(11) $$\Delta \varepsilon =\Delta \varepsilon^{e}+\Delta \varepsilon^{p}+\Delta \varepsilon^{\mathrm{tr}}+\Delta \varepsilon^{\mathrm{tp}}+\Delta \varepsilon^{T}$$

where $\Delta \varepsilon^{e}$, $\Delta \varepsilon^{p}$, $\Delta \varepsilon^{T}$, $\Delta \varepsilon^{\mathrm{tr}}$, and $\Delta \varepsilon^{\mathrm{tp}}$ are the strain changes induced by elastic deformation, plastic deformation, temperature change, transformation, and transformation plasticity.

For the test on a Gleeble thermal-mechanical simulator, the measured strain is longitudinal to the load application/current flow axis. Assuming that the stress is less than the yield strength during the martensitic transformation, then it can be written as [23]:

(12) $$\Delta \varepsilon^{l}=\Delta \varepsilon^{\mathrm{tr}}+\Delta \varepsilon^{\mathrm{el}}+\Delta \varepsilon^{\mathrm{tpl}}+\Delta \varepsilon^{T}=\Delta \varepsilon^{\mathrm{tr}}-\mu \Delta \varepsilon^{\mathrm{et}}-\frac{1}{2}\Delta \varepsilon^{\mathrm{tpt}}+\Delta \varepsilon^{T}$$

where $\Delta \varepsilon^{l}$, $\Delta \varepsilon^{\mathrm{el}}$, and $\Delta \varepsilon^{\mathrm{tpl}}$ are the measured strain, the elastic strain, and the transformation plasticity stain in the longitudinal direction to the load application/current flow axis. $\Delta \varepsilon^{\mathrm{et}}$ and $\Delta \varepsilon^{\mathrm{tpt}}$ are the elastic stain and the transformation plasticity stain in the transverse direction. μ is the Poisson ratio of specimens.

The change of transformation strain can be calculated by [4]:

(13) $$\Delta \varepsilon^{\mathrm{tr}}=\frac{\Delta V}{3V_{0}}f$$

where $V_{0}$ is the average atomic volume of austenite at the reference temperature, and ΔV is the difference between the average atomic volume of martensite and austenite.

Taking $M_{s0}^{\prime }$, the martensite start temperature under stress at the point with the relative position 0, as the reference temperature, the change of temperature change strain can be obtained by mixing law [23]:

(14) $$\Delta \varepsilon^{T}=\left[\beta^{\gamma }\left(1-f\right)+\beta^{m}f\right]\left(T-M_{s0}^{\prime }\right)=\left[\beta^{\gamma }-\left(\beta^{\gamma }-\beta^{m}\right)f\right]\left(T-M_{s0}^{\prime }\right)$$

where $\beta^{\gamma }$ and $\beta^{m}$ are the expansion coefficient of austenite and martensite.

According to Schuh and Dunand’s induction [24], the change of the transformation plasticity stain can be approximated as:

(15) $$d\Delta \varepsilon_{}^{\mathrm{tpt}}=\frac{5}{6}\frac{\Delta V}{V}\frac{\sigma_{1}}{\sigma_{Y}}df\approx \frac{5}{6}\left[\frac{\Delta V_{0}}{V_{0}}-3\left(\beta^{\gamma }-\beta^{m}\right)\left(T-M_{s}^{\prime }\right)\right]\frac{\sigma_{1}}{\sigma_{Y}}df$$

where ΔV/V is the volume mismatch between austenite and martensite, $\Delta V_{0}/V_{0}$ is the volume mismatch at the reference temperature, $\sigma_{Y}$ is the yield stress of the weaker phase, and $\sigma_{1}$ is the applied external stress.

Considering that most of the martensite is generated rapidly near $M_{s}$, the strain from transformation plasticity can be approximated as a linear function of the martensitic fraction. Then the change of transformation plasticity strain can be calculated by:

(16) $$\Delta \varepsilon^{\mathrm{tpt}}=\int_{0}^{f}\frac{5}{6}\left[\frac{\Delta V_{0}}{V_{0}}-3\left(\beta^{\gamma }-\beta^{m}\right)\left(T-M_{\mathrm{sx}}^{\prime }\right)\right]\frac{\sigma_{1}}{\sigma_{Y}}df\approx \frac{5}{6}\frac{\Delta V_{s}}{V_{s}}\frac{\sigma_{1}}{\sigma_{Y}}f$$

where $\Delta V_{s}/V_{s}$ is the volume mismatch at $M_{s}$.

During the martensitic transformation, the material parameters of the specimen change with the martensitic fraction. The Young′s modulus of the specimen can be expressed by [23]:

(17) $$K=\frac{K^{m}K^{\gamma }}{f\left(K^{\gamma }-K^{m}\right)+K^{m}}$$

where $K^{m}$ and $K^{\gamma }$ are Young′s modulus of the martensite and the austenite.

The strain due to elastic deformation can be calculated by Hooke’s law,

(18) $$\varepsilon^{\mathrm{et}}=\frac{\left(K^{\gamma }-K^{m}\right)f+K^{m}}{K^{m}K^{\gamma }}\sigma_{1}=\frac{\sigma_{1}}{K^{\gamma }}+\frac{K^{\gamma }-K^{m}}{K^{m}K^{\gamma }}\sigma_{1}f$$

The change of elastic strain can be calculated by:

(19) $$\Delta \varepsilon^{\mathrm{et}}=\frac{K^{\gamma }-K^{m}}{K^{m}K^{\gamma }}\sigma_{1}f$$

Combining Equations (12)–(14), (16), and (19), the strain change in the longitudinal direction has the following relationship with the martensitic fraction:

(20) $$\Delta \varepsilon^{l}-\beta^{\gamma }\left(T-M_{s}^{\prime }\right)=\left[\frac{\Delta V_{0}}{3V_{0}}-\left(\beta^{\gamma }-\beta^{m}\right)\left(T-M_{s}^{\prime }\right)-\mu \frac{\left(K^{\gamma }-K^{m}\right)}{K^{m}K^{\gamma }}\sigma_{1}-\frac{1}{2}\times \frac{5}{6}\frac{\Delta V_{s}}{V_{s}}\frac{\sigma_{1}}{\sigma_{Y}}\right]f$$

Since the term $\left(\beta^{\gamma }-\beta^{m}\right)\left(T-M_{s}^{\prime }\right)$ is much smaller than the other terms, Equation (20) can be simplified to:

(21) $$f=\frac{\Delta \varepsilon^{l}-\beta^{\gamma }\left(T-M_{s}^{\prime }\right)}{\frac{\Delta V_{0}}{3V_{0}}-\mu \frac{\left(K^{\gamma }-K^{m}\right)}{K^{m}K^{\gamma }}\sigma_{1}-\frac{5}{12}\frac{\Delta V_{s}}{V_{s}}\frac{\sigma_{1}}{\sigma_{Y}}}$$

Equation (21) reveals that the martensitic fraction is approximately linearly related to the difference between the measured strain and the strain due to temperature change.

### 2.3. The Determine of the Transformation Parameter α and the Martensite Start Temperature

The classic martensitic transformation kinetics model that has been widely applied was proposed by Koistinen and Marburger in 1959 [25]. In this study, the accurate fraction of retained austenite in different Fe–C alloys with 0.37 to 1.10 wt.% carbon was measured with an X-ray diffractometer and a fitted relationship was found, as follow:

(22) $$f=1-\exp\left[-\alpha \left(M_{s}-T\right)\right]$$

where *α* is a constant and equal to 0.011 for Fe–C alloy with less than 0.11 wt.% carbon.

Ignoring the difference between the transformation parameters *α* in the surface/edge zone and the core/middle zone, the martensitic fraction under a temperature gradient can be calculated using the following equations.

When $M_{s}\leq T\leq M_{s0}$, the martensitic fraction can be calculated by:

(23) $$f=f_{S}=2\int_{0}^{\frac{F_{S}}{2\Delta T_{0}}\left(T-M_{s0}\right)}\left\{1-\exp\left[-\alpha \left(M_{\mathrm{sx}}-T\right)\right]\right\}dx=\frac{F_{S}}{\Delta T_{0}}\left(T-M_{s0}\right)+\frac{F_{S}}{\alpha \Delta T}-\frac{F_{S}}{\alpha \Delta T}\exp\left[-\alpha \left(M_{s0}-T\right)\right]$$

where $f_{S}$ is the martensitic fraction of the surface/edge zone.

When $T\leq M_{s}$, the martensitic fraction can be calculated by:

(24) $$f=f_{S}+f_{C}=2\int_{0}^{\frac{F_{S}}{2}}\left\{1-\exp\left[-\alpha \left(M_{\mathrm{sx}}-T\right)\right]\right\}dx+\left(1-F_{S}\right)\left\{1-\exp\left[-\alpha \left(M_{s}-T\right)\right]\right\}=1-\exp\left[-\alpha \left(M_{s}^{g}-T\right)\right]$$

where $f_{C}$ is the martensitic fraction of the core/middle zone, $M_{s}^{g}$ is the equivalent transformation start temperature under a temperature gradient and can be expressed by:

(25) $$M_{s}^{g}=M_{s}-\frac{1}{\alpha }\ln\left\{-\frac{F_{S}}{\alpha \Delta T_{0}}\left[1-\exp\left(\alpha \Delta T_{0}\right)\right]+\left(1-F_{S}\right)\right\}=M_{s}-\frac{1}{\alpha }\ln\left(1-F_{M_{s}}\right)$$

where $F_{M_{s}}$ is the fraction of martensite at $M_{s}$.

**Figure 4:**
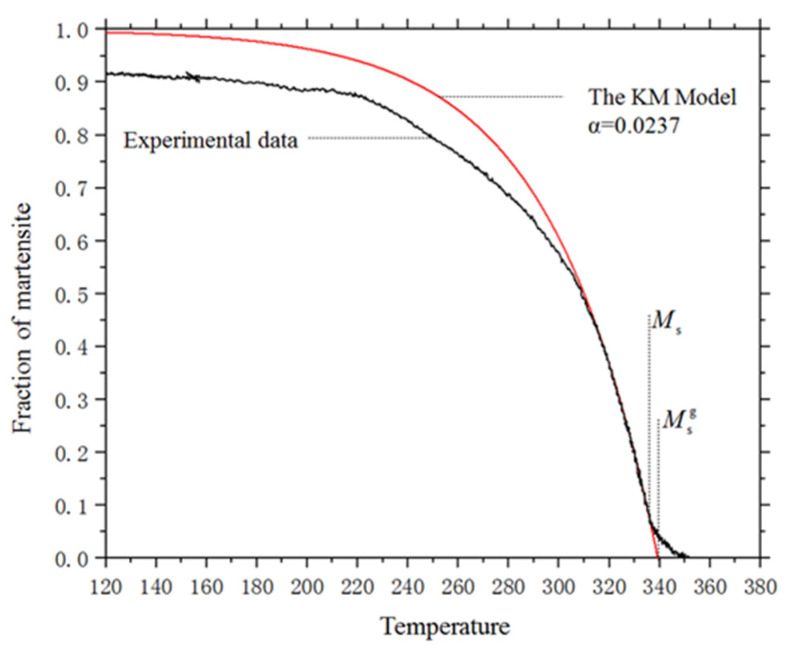
The experimental curve and the fit KM model.

Although the KM model was found to fit well with the experimental data only in the initial stage in many studies, according to Equations (23) and (24), the initial value of parameters *α* and $M_{s}^{g}$ can be determined by fitting the measured kinetics curve with the KM-equation when $T\leq M_{s}$ as shown in Figure 4. In addition, $M_{s}$ can be obtained by finding the highest temperature where the KM model coincides with the kinetic curve.

## 3. Experimental Procedure

**Figure 5:**
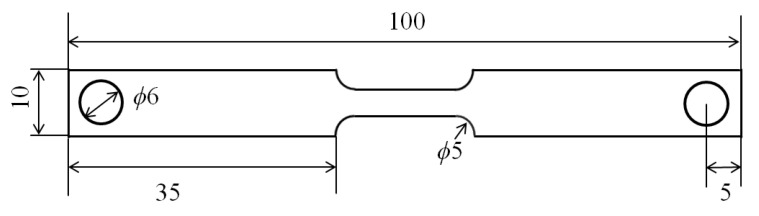
The drawing of specimens for dilatometric experiment.

The chemical composition of the studied low-carbon alloyed steel is shown in Table 1, which was measured using a Spectrolab M10 stationary metal analyzer. The specimens, from a cold-rolled sheet, with an original microstructure of ferrite and pearlite and a thickness of 1.8 mm, were cut into shape, as shown in Figure 5.

**Figure 6:**
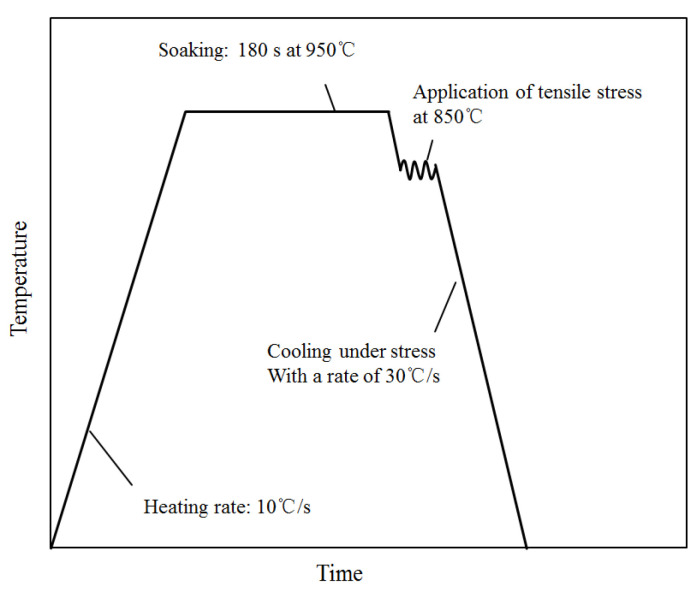
The thermo-mechanical processes of the dilatometric experiment.

The heating, quenching, and loading process was performed using a Gleeble-1500 thermal-mechanical simulator, and the applied stress in the experimental process was set according to Figure 6.

To obtain an initial complete, homogeneous austenitizing microstructure, the specimens were heated to the austenitizing temperature of 950 °C with a rate of 10 °C/s and had a soaking time of 3 min. As shown in Figure 6, the specimens were cooled to 850 °C at a rate of 30 °C/s after soaking. Then a constant tensile stress was put on the specimens. With the constant stress, the specimens were quenched to room temperature, with a cooling rate of 30 °C/s.

## 4. Results and Discussion

### 4.1. The Kinetics of Martensitic Transformation without External Stress

**Figure 7:**
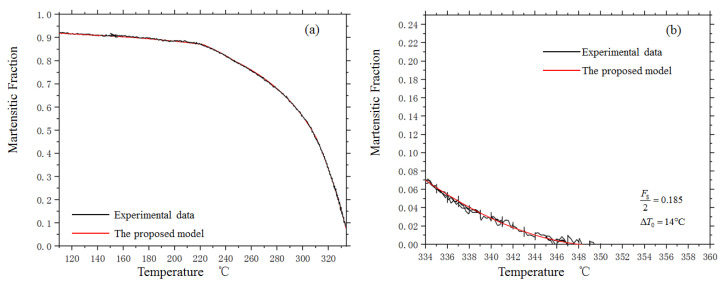
The kinetics curve of martensitic transformation under temperature gradient. (**a**) The kinetics curve when $T\leq M_{s}$ (**b**) The kinetics curve when $M_{s}\leq T\leq M_{s0}$.

Figure 7 shows the measured dilatometric curves of the investigated low-alloy steel under a temperature gradient without external stress. Although the martensitic kinetic curves of many kinds of steel, including the investigated steel, have a similar shape to the KM model, it is important to note that, according to many studies, the parameter *α* is only constant in the middle stage of the transformation kinetics curve, between 5% and 60% martensite, and changes in the initial stage and the ending stage [26].

Through experimental observation, Magee [27] derived the thermodynamic form of the KM-model. The newly formed number of martensite laths d*N* and the change of the driving force d*U* have the following proportional relationship:

(26) dN=ϕdU

Then the fraction of the newly formed martensite can be expressed by:

(27) $$df=\bar{V}dN=\phi \bar{V}\frac{d\Delta G^{\gamma \to \alpha }}{dT}\left(1-f\right)dT$$

where $\bar{V}$ is the average volume of newly formed martensitic laths.

The integral calculation gives Equation (22), and *α* can be expressed as:

(28) $$\alpha =-\frac{d\Delta G^{\gamma \to \alpha }}{dT}\phi \bar{V}$$

The volume of martensitic laths is constrained by the grain boundary and the formed laths and changes gradually during transformation, which means *α* is not always constant during martensitic transformation. Therefore, it is reasonable to improve the KM-model as following:

(29) $$f=1-\exp\left[-\alpha_{F}\left(M_{s}-T\right)\right]$$

where $\alpha_{F}$ is a parameter that is constant at the beginning of the transformation but changes with the fraction of the formed martensite in the following stages.

According to Equation (29), the parameter $\alpha_{F}$ can be extracted from the measured kinetics curve by:

(30) $$\alpha_{F}=-\frac{1}{M_{s}^{g}-T}\ln\left(1-f\right)$$

**Figure 8:**
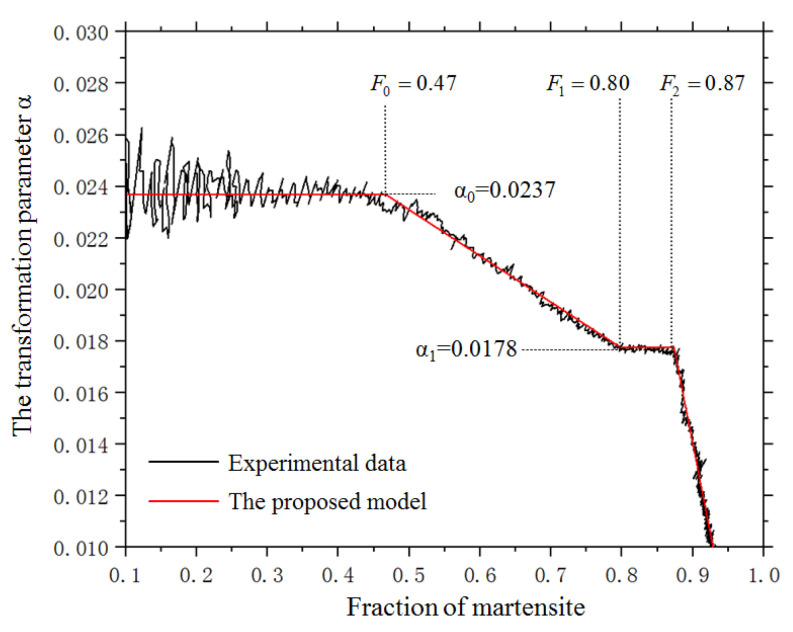
The measured parameter $\alpha_{F}$ during the transformation without stress.

Figure 8 shows the parameter $\alpha_{F}$ as a function of the formed martensite fraction. It indicates that $\alpha_{F}$ is constant in the first half of the transformation, where the transformed martensite is less than 47%. Then, $\alpha_{F}$ enters a linearly decreasing stage until 80% of austenite has transformed into martensite. Beyond this stage, $\alpha_{F}$ becomes a constant once more and equals approximately one-quarter of the initial value. When *f* is more than 87%, $\alpha_{F}$, starts to decrease linearly again, with a higher rate.

Through fitting, $\alpha_{F}$ can be expressed as:

(31) $$\alpha_{F}=\left\{ \begin{array}{l} \alpha_{0}\;\left(0\leq f\leq 0.47\right)\\ \alpha_{0}\left[1-0.7353\left(f-0.46\right)\right]\;\left(0.47<f<0.8\right)\\ 0.75\alpha_{0}\;\left(0.8\leq f\leq 0.87\right)\\ 0.75\alpha_{0}\left[1-7.692\left(f-0.87\right)\right]\;\left(0.87\leq f\leq 1\right) \end{array}\right.$$

for the tested steel, $\alpha_{F}$ equals 0.0237.

When $T<M_{s}$, according to Equations (29) and (31), the kinetics curve of martensitic transformation without stress can be expressed as:

(32) $$T=M_{s}+\frac{1}{\alpha_{F}}\ln\left(1-f\right)$$

The proposed kinetics model shows a good agreement with the experimental curve, as shown in Figure 7a. Considering that the turning points of the parameter $\alpha_{F}$ are only dependent on geometric constraints, it can be concluded that the proposed model applies to all the lath martensitic transformations with close habit planes and sliding directions.

In Figure 7b, the agreement between the experimental data and the model prediction confirms that the deviation between the experimental data and the previous kinetics models in the initial stage comes from the effect of the temperature gradient.

### 4.2. The Kinetics of Martensitic Transformation under Stress

**Figure 9:**
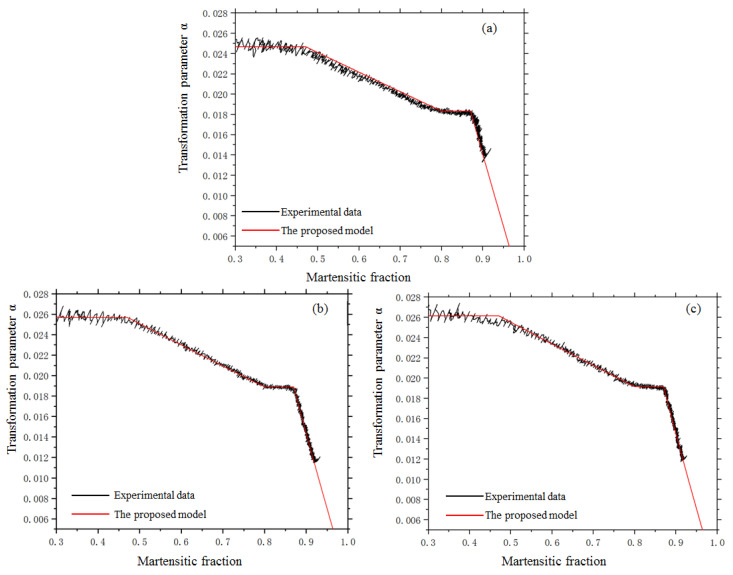
The parameter $\alpha_{F}^{\prime }$ during the transformation under stress: (**a**) 32.7 MPa. (**b**) 65.4 MPa. (**c**) 81.7 MPa.

Figure 9 shows the parameter $\alpha_{F}$ as a function of the martensitic fraction under a temperature gradient and external stress. Although the curves under different stresses show the same pattern of variation, the values of $\alpha_{F}$ under stress are bigger than the value without stress and increase with stress.

Considering the mechanical driving energy from stress, according to Equation (26), the fraction of the newly formed martensite can be expressed by the following equation, when $T\leq M_{s}$:

(33) $$df=\phi \bar{V}\left[\frac{d\Delta G^{\gamma \to \alpha }}{dT}\left(1-F_{M_{s}}^{\prime }-f\right)dT+\bar{U^{\prime }}\right]=\phi \bar{V}\left[\frac{d\Delta G^{\gamma \to \alpha }}{dT}\left(1-F_{M_{s}}^{\prime }-f\right)dT+\varphi \sigma_{1}df\right]$$

where $\bar{U^{\prime }}$ is the average mechanical driving force from the applied stress. φ is a constant. $F_{M_{s}}^{\prime }$ is the fraction of martensite induced by stress at $M_{s}$.

The integral calculation gives an approximate expression for the martensitic fraction:

(34) $$f=1-\exp\left[-\alpha_{F}^{\prime }\left(M_{s}^{\mathrm{st}}-T\right)\right]$$

where $M_{s}^{\mathrm{st}}$ is the equivalent transformation start temperature under stress and can be expressed by:

(35) $$M_{s}^{\mathrm{st}}=M_{s}-\frac{1}{{\alpha^{\prime }}_{F}}\ln\left(1-\alpha_{F}^{\prime }\right)$$

$\alpha_{F}^{\prime }$ is the transformation parameter under stress and can be expressed by:

(36) $$\alpha_{F}^{\prime }=\frac{1}{1-\phi \bar{V}\varphi \sigma_{1}}\alpha_{F}\approx \alpha_{F}\left(1+\phi \bar{V}\varphi \sigma_{1}\right)=\alpha_{F}\left(1+\psi \alpha_{F}\sigma_{1}\right)$$

where ψ is a constant.

Approximately, assuming that the parameter $\alpha_{F}^{\prime }$ in the surface/edge zone is always equal to $\alpha_{F}^{\prime }$ in the core/middle zone, when $T<M_{s}$, the total fraction of formed martensite can be obtained by:

(37) $$\begin{array}{ll} f & =f_{S}+f_{C}\\ & =2\int_{0}^{\frac{F_{S}}{2}}\left\{1-\exp\left[-\alpha_{F}^{\prime }\left(M_{s}^{\mathrm{st}}-T\right)\right]\right\}dx+\left(1-F_{S}\right)\left\{1-\exp\left[-\alpha_{F}^{\prime }\left(M_{s}^{\mathrm{st}}-T\right)\right]\right\}\\ & =1-\exp\left[-\alpha_{F}^{\prime }\left(M_{s}^{\mathrm{st}\text{-}g}-T\right)\right] \end{array}$$

where $M_{s}^{\mathrm{st}\text{-}g}$ is the equivalent transformation start temperature under stress and can be calculated by:

(38) $$M_{s}^{\mathrm{st}\text{-}g}=M_{s}-\frac{1}{\alpha_{F}^{\prime }}\ln\left\{\left(1-F_{M_{s}}^{\prime }\right)\left\{\frac{F_{S}}{\alpha_{F}^{\prime }\Delta T_{0}}\left[1-\exp\left({\alpha^{\prime }}_{F}\Delta T_{0}\right)\right]+\left(1-F_{S}\right)\right\}\right\}=M_{s}-\frac{1}{\alpha_{F}}\ln\left(1-F_{M_{s}}\right)$$

According to Equation (34), $\alpha_{F}^{\prime }$ can be extracted from the measured kinetics curve by:

(39) $$\alpha_{F}^{\prime }=-\frac{1}{M_{s}^{\mathrm{st}\text{-}g}-T}\ln\left(1-f\right)$$

The parameter $\alpha_{F}^{\prime }$ under stress is shown in Figure 9 and can be expressed by the following equation through fitting:

(40) $$\alpha_{F}^{\prime }=\alpha_{F}\left(1+5.45\times 10^{-8}\alpha_{F}\sigma_{1}\right)$$

When $T<M_{s}$, according to Equations (34) and (40), the kinetics curve of martensitic transformation under stress can be expressed as:

(41) $$T=M_{s}+\frac{1}{\alpha_{F}\left(1+5.45\times 10^{-8}\alpha_{F}\sigma_{1}\right)}\ln\left(1-f\right)$$

## 5. Conclusions

Based on the proportional relationship between the martensitic fraction and the difference of the measured strain from thermal strain under stress, a new dilatometric analysis model was suggested to extract the kinetics curves and determine the transformation parameters. According to the dilatometric analysis results under different stresses, the KM kinetics model was refined and the improved model showed excellent agreement with the experiment results. Furthermore, the following conclusions can be drawn.

(1) The parameter $\alpha_{F}$ was not a constant but a variable, expressed as a segmentation function with the martensitic fraction as the independent variable. This phenomenon can be attributed to the linear relationship between α and the average volume of newly formed martensitic laths.

(2) As a part of the driving force of martensitic transformation, the mechanical energy from stress increased the value of $\alpha_{F}$ linearly.

## Figures and Tables

**Table 1:** Chemical composition (wt.%) of the investigated steel.

C	Mn	Si	B	Cr	Ti	P	Ni	Al	Cu	Mo	Co	S
0.360	1.240	0.232	0.002	0.116	0.031	0.012	0.018	0.016	0.011	0.005	0.005	0.002

## Data Availability

Data supporting the findings of this study are available from the corresponding author upon request.
